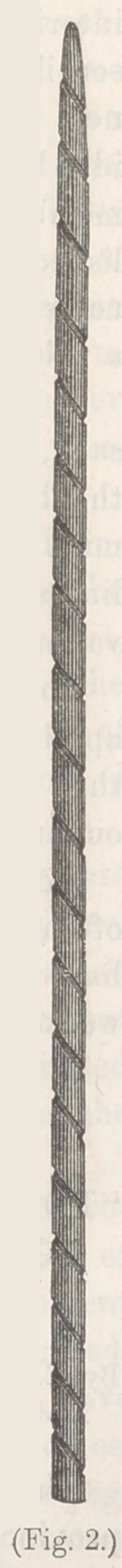# New Methods of Treating Urethral Diseases

**Published:** 1868-09

**Authors:** J. S. Sherman

**Affiliations:** Chicago


ARTICLE XXXII.

### NEW METHODS OF TREATING URETHRAL DISEASES.

BY J. S. SHERMAN, M.D., Chicago.

The frequent failure of the ordinary treatment pursued in urethral inflammations and the continuance of many blennorrhœas, not only for months, but of years, has shown the insufficiency of the old methods. There is in these diseases, as in all others, a strong tendency to spontaneous recovery; yet there are several satisfactory reasons why inflammations of the urethra so often resist treatment:

1st. The disease is in a position where it is difficult to make the necessary local applications. The attempt to accomplish this by means of injections is very insufficient; the fluid remains but a short time in contact with the diseased surfaces, and it is seldom that the patient or surgeon succeeds in carrying it more than one-half the depth of the canal, the accelerator urinæ muscle preventing its further progress. The internal administration of copaiba, cubebs, and the terebinthinate preparations, with the hope of influencing the disease by the contact of urine impregnated with these remedies, is so often followed by severe gastric disturbance, that the patient prefers the disease to the treatment; and in the chronic cases of urethritis these remedies are useless.

2d. The constant contact of the inflamed walls of the urethra, bathed in specific pus, is not only a cause of the rapid extension of the malady, but also of its pertinacity and obstinacy. The poison is ever present in the pus, and the inflammation aggravated by the contact of the diseased surfaces. These conditions are certainly not overcome by injections or internal administration of drugs.

3d. There is a tendency in the mucous membrane of the urethra to develop granulations similar to those formed on the eyelids. The histological character of the urethral membrane is very similar to the conjunctiva of the eye, and gonorrhœal inflammations of these tissues frequently terminate in granulations. This fact was first discovered and made known by M. Desormeaux, who, by the aid of his endoscope, was enabled to demonstrate them in some cases.

From these conditions, the indications for treatment are plain: the destruction of the specific character of the pus, and complete separation from contact of the diseased surfaces, being the most important. Astringent effect is valuable in diminishing vascularity; yet if the first two indications are successfully met, the vascularity and consequent production of pus will rapidly subside.

For the means and manner of applying remedies directly to the urethra, in such a way that they remain long in contact with every portion of the canal, we are indebted to the ingenuity of M. Mallez. His special attention has for several years been devoted to genito-urinary diseases, and the results of his study are given to the profession in the first volume of his "Album d'Anatomie Pathologique," containing photographic representations of these diseases, taken directly from dissections. From personal observation of cases treated by him, I have no hesitation in saying that better results are obtained by his mode than I have seen follow any other plan.

(Fig. 1.)

(Fig. 1.) represents an instrument for applying remedies, in the form of powders, to the urethra. It consists of the following parts: *a*, a small rubber sack, terminating in a metal tube, which is provided with a valve at the point *b* opening inwards and allowing air to enter, but closing when the sack is compressed; *c*, a scoop, which is received in the larger tube and terminates in a small elastic catheter; *d*, a large elastic catheter, into which the smaller one is easily passed, leaving a space between the two. The larger should project somewhat beyond the smaller. It is thus used: The large catheter is first introduced into the urethra, beyond the point of disease, and being elastic, it can be passed into the prostatic portion if necessary. The secretions which have accumulated in the tube are removed with a pledget of cotton. The powder to be applied is then placed in the scoop, and connected with the sack. The small catheter is passed into the larger one and the sack compressed. This carries a current of air through the instrument, and with it the powder, which is deposited on the mucous membrane, at its termination. If the instrument be slowly removed, at the same time that the insufflations are made, the whole interior of the canal will receive a coating of the powder, and portions which could not be reached with injections will be effectually acted upon. The powders must be blown with considerable force, in order that they may impinge strongly on the mucous surface.

The remedies used must be finely powdered, and will vary according to circumstances. The sub. nit. bismuth is very valuable, and often of itself proves sufficient. It adheres firmly to the membrane, and forms a coating which effectually separates the diseased surfaces. The following will be found applicable to a majority of cases:

℞. Potas. Permanganate, ............. gr. j.
Quiniæ Tannate, ............. gr. vj.
Bismuth Sub. Nit., ............. ℥ij.

M. S. Use as an insufflation, once daily.

Various other remedies may be used, as tannin, sulph. copper, zinc, etc. The only caution necessary is, that the powders be not used too strong. When the disease is produced by stricture, dilation offers the only chance of cure.

Remedies which cannot be used in the form of powders, may be applied as ointments, by means of the spiral bougie, shown in (Fig. 2.) It is made of wax, and has the sur-

(Fig. 2.)

face marked with a spiral groove, into which the ointment to be used is placed.

The local application of carbonic acid gas to the urethra and interior of the bladder is a valuable means of allaying morbid sensibility. Spermatorrhœa, when accompanied with tenderness in the vicinity of the ejaculatory ducts, is frequently rapidly benefited by its use. It is a powerful sedative to mucous membranes. Its reputation in gastric irritation is well established, and it can be applied to the urethra and bladder effectually and with great benefit. In vesical catarrh it will be found a valuable remedy.

A simple way of using it is to collect the gas in a rubber sack, to which is attached a tube that can be introduced into the bladder. The gas is then forced in by pressure. Care must be taken that we do not distend the organ beyond proper limits. The same principles of treatment are applicable in vaginal diseases of the female.

Powdered substances have, in the manner described, been applied to the dead subject, and dissection has shown the urethra completely covered with the substance, proving the thoroughness of the application.

Acute cases are very promptly cured, and the discharge is often immediately arrested. The old and chronic ones, which have lasted for years, require time, varying from three to six weeks.

## Figures and Tables

**Fig. 1. f1:**
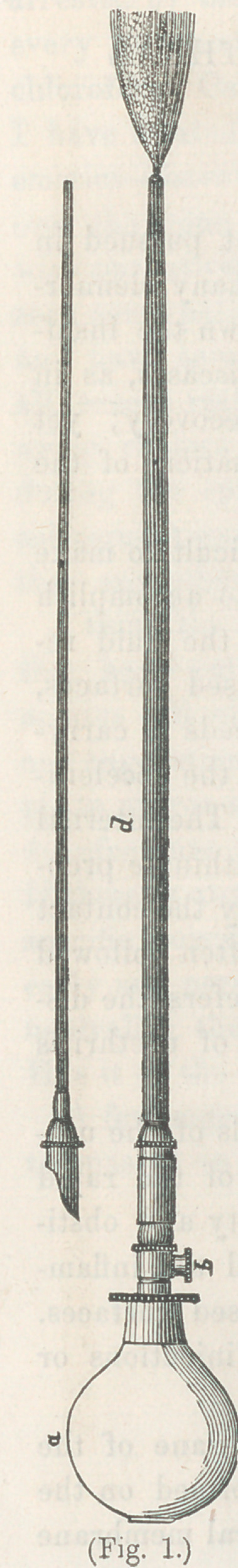


**Fig. 2. f2:**